# Psychological and social difficulties in young non-help-seeking adolescents at risk for psychosis: insights from a large cohort study

**DOI:** 10.3389/fpsyg.2024.1430805

**Published:** 2024-09-17

**Authors:** Charlotte M. Teigset, Christine Mohn, Caroline R. Mohn-Haugen, Frank Larøi, Bjørn Rishovd Rund

**Affiliations:** ^1^Vestre Viken Hospital Trust, Drammen, Norway; ^2^Institute of Clinical Medicine, Faculty of Medicine, University of Oslo, Oslo, Norway; ^3^Department of Psychology, University of Oslo, Oslo, Norway

**Keywords:** adolescents at-risk, non-help-seeking, self-reported psychosis symptoms, depression, anxiety, self-esteem, social function, psychological difficulties

## Abstract

**Background:**

This study used data from the Norwegian Mother, Father, and Child Cohort Study (MoBa), and explored the psychological and social challenges of 14-year-olds who report psychotic symptoms. Research on help-seeking youths indicates comorbid symptoms of depression, anxiety, and social deterioration, but less is known about non-help-seeking individuals who may not use healthcare services, possibly skewing comorbidity profiles. Also, findings suggest that adolescents manifesting psychotic symptoms refrain from pursuing help. This gap underscores the necessity of studying non-help-seeking adolescents to better understand their needs and the risks they face without intervention.

**Methods:**

We analyzed responses from adolescents who completed the 14-year questionnaire in MoBa (*N* = 127), identifying those as at risk by their high scores on psychosis-risk items, within the top 0.4% (*N* = 58). Comparative analyses were conducted against matched controls to assess differences in psychological and social functioning (*N* = 69).

**Results:**

Results indicated that the at-risk adolescents experience significantly more depression and anxiety and have lower self-esteem and poorer social functioning than controls. Social functioning parameters, including leisure activities, social competence, quality of parental relationship, and sense of school belonging, were significantly worse than those observed in controls. The results indicate a pronounced vulnerability among non-help-seeking adolescents at-risk, similar to issues seen in help-seeking youths.

**Conclusion:**

These findings highlight the importance of early identification and intervention strategies that reach beyond traditional clinical settings, suggesting the efficacy of population or community-based screenings to prevent long-term adverse outcomes. The study proposes a broader understanding of psychosis risk, stressing the importance of inclusive approaches to support at-risk adolescents effectively.

## Introduction

Psychosis involves a major personal burden, impacting relationships and overall well-being, as well as leading to substantial societal costs through lost productivity and increased healthcare demands (Jongsma et al., 2019). Studies of adolescents at risk for psychosis are pivotal for understanding the nascent phases of psychotic disorders. Early detection, understanding disease progression, and recognizing initial symptoms enable interventions that can lessen severity, delay onset, or potentially prevent psychosis (Radez et al., 2023; Fusar-Poli et al., 2013). Evidence suggests that nearly half of all mental health disorders commence by the age of 14 (Colizzi et al., 2020), indicating that early adolescence is particularly crucial. However, research remains sparse (Lo Buglio et al., 2022), and with a predominant focus on help-seeking adolescents and young adults at clinical high risk (CHR) for psychosis. These youths are characterized by symptoms ranging from subthreshold psychotic experiences to genetic vulnerabilities toward psychosis, compounded by a deterioration in functional capacity (Addington et al., 2017; McGlashan et al., 2010).

Youth at CHR often experience concurrent psychological and social difficulties that not only amplify the subjective experience of their psychotic symptoms but that also are prognostic of poorer long-term outcomes (Wigman et al., 2012; Fusar-Poli et al., 2014; Heinze et al., 2018). Research indicates that they are more vulnerable for depression, anxiety, cannabis use, and experience significant impairments in social functioning, self-reliance, parental relationships, as well as reductions in quality of life, self-esteem, and self-efficacy (Addington et al., 2017; Fusar-Poli et al., 2014; Heinze et al., 2018; Woods et al., 2009; Salokangas et al., 2012; Rusch et al., 2014; Addington et al., 2021; Ballon et al., 2007; Shim et al., 2008; Braun et al., 2022; Devoe et al., 2019; Thompson et al., 2015; Cleverley et al., 2024; Solmi et al., 2023; McAusland et al., 2017). A meta-analysis encompassing 1,684 CHR youths reported prevalences of anxiety and depression at 15 and 41%, respectively (Fusar-Poli et al., 2014). In the general population, it is estimated that 3.6% of 10–14-year-olds and 4.6% of 15–19-year-olds have an anxiety disorder, while depression occurs in 1.1% of 10–14-year-olds and 2.8% of 15–19-year-olds (WHO, n.d.). This emphasizes the substantial degree of comorbidity in CHR youths.

The referenced CHR research has provided important knowledge, however, the studies have predominately included older adolescents and young adults [mean-ages of 15–29 years in Fusar-Poli et al. (2014)]. Considering the rapid emotional and social development characteristic of early adolescence (Blakemore, 2019; Yoon et al., 2023), psychotic and comorbid symptoms may be experienced differently than later in life. Therefore, it is imperative for research to include young adolescents, as well as account for age-related variations in symptomatology (Schultze-Lutter et al., 2020).

In addition, the referenced CHR research exclusively included help-seeking individuals. CHR individuals are recruited from healthcare providers, which may bias the comorbidity profiles: those with comorbid symptoms may be more inclined to seek professional help and participate in research, whereas those without such symptoms may not (Addington et al., 2017). Moreover, many adolescents manifesting psychotic symptoms refrain from pursuing help (Nordgaard et al., 2021; Allan et al., 2021). Thus, studies including non-help-seeking adolescents are urgently needed.

These limitations are partly met by CHR research concentrating on children and young adolescent groups. The findings indicate high levels of anxiety, depression (Lo Buglio et al., 2022), and impairments in social functioning (Tikkanen et al., 2022; Tikkanen et al., 2019). High levels of depression and anxiety are also detected in adolescents with psychotic-like experiences (PLEs), that is, psychotic experiences that are less intense and distressing than those found in CHR populations (WHO, n.d.; Stainton et al., 2021; Wu et al., 2021). However, to reach non-help-seeking adolescents with psychotic symptoms, the deployment of self-report instruments within educational or community settings presents an efficient and cost-effective strategy (Radez et al., 2023; Allan et al., 2021; Howie et al., 2020; Fusar-Poli et al., 2020). Such self-reports correlate highly with clinical evaluations (Thompson et al., 2014), and one conducted study, however, still including young adults (13–21 years), found that students who disclosed psychotic symptoms also reported emotional, behavioral, and social challenges (Fonseca-Pedrero et al., 2017). Another study, encompassing 13-year-olds who had described psychotic symptoms 3 months prior, leveraged data from the Avon Longitudinal Study of Parents and Children Cohort, and correlated parent-reported peer-related challenges with poorer social functioning (Asher et al., 2013). The findings of deteriorated social functioning were attributed to emotional challenges, suggesting that the absence of emotional problems may preserve social functioning despite psychotic symptoms (Asher et al., 2013). This study relied on parental reports, leaving questions about the adolescents self-perceived difficulties, especially in comparison to peers not experiencing psychotic distress.

The current study aimed to explore psychological and social difficulties in adolescents at risk for psychosis, specifically addressing two gaps in previous knowledge by including: (if) a group of young adolescents, and (ii) those who are non-help-seeking. By deriving data from the 14-year survey in The Norwegian Mother, Father, and Child Cohort Study (MoBa), our objective was to explore whether the adolescents who report psychotic symptoms also, in comparison to matched controls without psychotic distress, reported psychological and social difficulties, as indicated by measures of depression, anxiety, self-esteem, and social functioning.

## Methods

### The Norwegian Mother, Father, and Child Cohort Study

The current study is conducted by the Vestre Viken Hospital Trust in Norway and is a sub-study of MoBa (Magnus et al., 2016). The following description is mandatory in all MoBa manuscripts: “*The Norwegian Mother, Father, and Child Cohort Study (MoBa) is a population-based pregnancy cohort study conducted by the Norwegian Institute of Public Health. Participants were recruited from all over Norway from 1999–2008. The cohort includes approximately 114,500 children, 95,200 mothers and 75,200 fathers, and 41% of all eligible pregnant women agreed to participate. The current study is based on MoBa data files released for research in March of 2024. The establishment of MoBa and initial data collection was based on a license from the Norwegian Data Protection Agency and approval from The Regional Committees for Medical and Health Research Ethics. The MoBa cohort is currently regulated by the Norwegian Health Registry Act. The current study was approved by The Regional Committees for Medical and Health Research Ethics in 2017 (ref. 2017/342)”* (MoBa, 2024).

### Participants

Previous reports provide a detailed description of the procedures (Mohn-Haugen et al., 2022; Mohn-Haugen et al., 2023). MoBa’s 14-year survey invited adolescents to self-report on 200 items related to psychological, physical, and social health (MoBa, 2022). The survey included 19 items focused on psychotic experiences and anomalous self-experiences (ASE), derived from the Community Assessment of Psychic Experiences questionnaire (CAPE-42) (Stefanis et al., 2002) and the Examination of Anomalous Self-Experience (EASE) (Parnas et al., 2005). These instruments are validated for identifying psychotic symptoms predictive of later psychotic disorders, also among adolescents. Specifically, the CAPE-42’s 16 items, including a subset from the CAPE-15, and the three ASE items crafted by EASE experts, allow for the assessment of symptom distress and impact (Bukenaite et al., 2017; Nelson et al., 2012; Jaya et al., 2021; Nunez et al., 2021; Sengutta et al., 2021; Vaernes et al., 2019; Svendsen et al., 2018). All 19 items are fully described in the MoBa Questions Documentation (MoBa, 2022), on page 32 for EASE and page 40 for CAPE.

**Figure 1:**
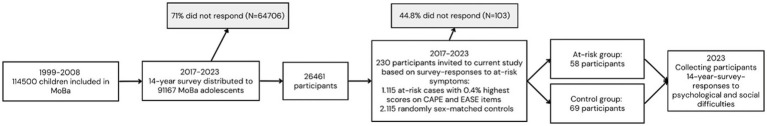
Flowchart of inclusion and data collection.

A flowchart detailing the inclusion criteria and data collection process is presented in Figure 1. The 14-year survey from the Norwegian Mother, Father and Child Cohort Study (MoBa) was distributed to 91,167 adolescents, with a 29% response rate (*N* = 26,461). For the present sub-study, 230 adolescents from the MoBa survey were selected based on their responses to 19 at-risk items and were subsequently invited to participate. This cohort comprised 115 individuals with scores in the top 0.4% on these items and 115 matched controls whose scores did not fall within the top 0.4% range. Among the high-risk group, 50% (*N* = 58) agreed to participate, while 60% (*N* = 69) of the controls consented to participation.

The at-risk group was precisely defined as the top 0.4% scorers on the CAPE and EASE items (MoBa, 2022), differentiating them from CHR or ultra-high-risk individuals typically identified through clinical interviews. The strict cut-off was strategically determined in collaboration with The Regional Committee for Medical and Health Research Ethics in Norway. This decision was based on discussions with experts in high-risk studies and preliminary analyses of survey data, which indicated the number of participants achieving full or near-full scores on the 19 selected items. A major concern was to avoid including false positives and to target only the adolescents experiencing frequent and distressing symptoms, characterized here as “at risk for psychosis.” The control group was randomly selected and matched by age and sex. Invitations to participate were sent to both adolescents and parents, including a detailed study description and consent form. Given the participants’ age (<16), parental consent was required. In addition to significant differences between the groups in terms of CAPE and EASE scores (due to the selection criteria), the two groups also differed significantly in terms of IQ (*p* < 0.01). Demographic and background details are summarized in Table 1.

**Table 1:** Group differences in demographic and background variables.

	At-risk *N* = 58	Controls *N* = 69	Cohen’s *d*	*p*
Sex (female/male)	51/7	61/8		NS
Age: mean (SD)	14.4 (0.5)	14.4 (0.5)		NS
Mothers’ education: mean (SD)	14.7 (2.5)	15.4 (2.2)		NS
WASI IQ 4 tests: mean (SD)	99.9 (10.3)	105.4 (11.0)	*d* = 0.52	0.004**
EASE 3 items: mean (SD)	2.7 (0.4)	1.4 (0.5)	*d* = 2.65	<0.001***
CAPE 16 items: mean (SD)	2.9 (0.3)	1.4 (0.3)	*d* = 4.87	<0.001***

**p < 0.01.

***p < 0.001.

NS, no significant difference between groups.

### Psychological and social assessments

The adolescents provided information on psychological and social functioning through self-reported responses to 11 different scales of the 14-year questionnaire, encompassing depression, anxiety, self-esteem, and social function. Full details and links to all tests are available in the MoBa Question Documentation (MoBa, 2022).

**Depression** assessment incorporated two tools: the Short Mood and Feelings Questionnaire (SMFQ) (Angold et al., 1995), and a subset of the Symptoms Checklist (SCL-10) specific to depression (Tambs and Røysamb, 2014).

Anxiety was measured through a section of the SCL-10 related to anxiety (Tambs and Røysamb, 2014), the Mini Social Phobia Inventory (Mini-SPIN) (Connor et al., 2001; Seeley-Wait et al., 2009) for assessing social anxiety, and the 5-item version of the Screen for Child Anxiety Related Disorders (SCARED) (Birmaher et al., 1997; Birmaher et al., 1999).

**Self-Esteem** was measured using four items from the Rosenberg Self-Esteem Scale (RSES) (Rosenberg, 1965; Rosenberg, 1986), a well-established instrument for assessing global self-esteem, including in adolescents. The selected items represent a concise version correlating strongly with the full scale (Tambs, 2004).

**Social Functioning** was assessed using four distinct tests focusing on leisure activities, social competence, relationships with parents, and school belonging. Leisure activities encompassed both physical and social activities, including homework and gaming, with physical activity items sourced from Sagatun et al. (2007). Social competence was evaluated through five questions adapted from the Self-perception Profile for Adolescents (Harter, 2012; Harter, 1988), revised and validated for a broader adolescent age range (Strand and von Soest, 2008; Wichstraum, 1995). Parental relationship quality was measured using four items from the Parental Relations Self-concept Scale (Marsh et al., 2005), and school belonging was determined through five items from the Norwegian PIRLS 2016 questionnaire, linked to academic and socio-emotional outcomes (Thapa et al., 2013; Wang and Degol, 2016).

This multi-faceted approach allowed for a comprehensive assessment of the adolescents’ psychological and social functioning, providing valuable insights into the four areas of concern.

### Analyses

Analyses were performed using SPSS. Two separate analyses were conducted.

First, we calculated four total scores for dimensions of psychological and social function by summarizing the scores within each category of depression, anxiety, social function, and self-esteem, resulting in four composite variables for each participant: Global Depression (GD), Global Anxiety (GA), Global Self-Esteem (GSE), and Global Social Function (GSF). Higher GD and GA scores indicated greater symptoms of depression and anxiety, while higher GSE and GSF scores implied better reported self-esteem and social functioning.

Secondly, unlike the three other composite scores, GSF included four tests measuring different domains of the adolescents social functioning. To explore differences in social function in more detail, we summarized scores from the reported symptoms in each domain of social function, giving us four separate social function variables: leisure time, social competence, quality of parental relationship, and a sense of school belonging.

Due to the small sample size, we first conducted two multivariate analyses of variance (MANOVA). In Analysis 1, the dependent variables included GD, GA, GSE, and GSF. The multivariate test (Wilks’ lambda) was significant (*F* = 24.65, *p* < 0.001, *η*^2^ = 0.52). In Analysis 2, the dependent variables included leisure time, social competence, quality of parental relationship, and school belonging. Similarly, the multivariate test (Wilks’ lambda) was significant (*F* = 10.42, *p* < 0.001, *η*^2^ = 0.30).

To assess whether the experienced psychological and social challenges (GD, GA, GSE, and GSF) differed significantly between the at-risk group and the sex- and age-matched controls, an independent samples *t*-test was performed. The scores of the at-risk group and the control group were sufficiently normally distributed for the purposes of conducting a *t*-test on all four measures (GD, GA, GSE, GSF) (i.e., skewness < 2.0 and kurtosis < 9.0; Schmider et al., 2010).

To determine if certain areas of GSF were more affected in the at-risk group compared to the controls, a new *t*-test for independent samples was conducted. This test evaluated the overall scores across each of the four GSF domains: leisure time, social competence, quality of parental relationship, and school belonging. As in the previous analysis, the at-risk group and the control group were sufficiently normal for the purposes of conducting a *t*-test on all four measures. To reduce the possibility of multiple testing influencing the results, the alpha levels for the second test was set at a stricter level of *p* < 0.01.

## Results

When analyzing whether the experienced psychological and social challenges (GD, GA, GSE, and GSF) differed significantly between the at-risk group and the sex- and age-matched controls, the results of the independent samples *t-*test were associated with statistically significant differences between the at-risk group and the controls on all the four measures of psychological and social difficulties. The effect sizes for the difference between groups were high, indicating large effects according to Cohen’s conventions (Table 2).

**Table 2:** Comparison of social functioning, self-esteem, depression and anxiety between adolescents at-risk for psychosis and healthy controls.

	At-risk *N* = 58	Controls *N* = 69				
	*M* (SD)	*M* (SD)	*t*	*p* ^5^	Cohen’s *d*	CI
Global social functioning^1^	11.1 (1.7)	13.2 (1.6)	6.35	<0.001***	*d* = 1.26	[95% CI: 0.83, 1.69]
Global self-esteem^2^	7.3 (2.6)	11.7 (2.8)	8.98	<0.001***	*d* = 1.60	[95% CI: 1.20, 2.00]
Global depression^3^	5.7 (1.0)	3.5 (1.2)	−9.81	<0.001***	*d* = −1.90	[95% CI: −2.35, −1.44]
Global anxiety^4^	8.5 (2.2)	5.1 (1.8)	−9.00	<0.001***	*d* = −1.71	[95% CI: −2.14, −1.27]

All global measures are composite test scores – average scores on included questions:

1 Global Social Function: selected items about leisure time, social competence (Self-Perceptions Profile for adolescents), parental relationship quality (Parental Relations Self-concept Scale), and school belonging (Norwegian PIRLS 2016).

2 Global Self-Esteem: Rosenberg Self-Esteem Scale (RSES).

3 Global Depression: The short Mood and Feelings Questionnaire, and the Symptom Checklist (SCL-10).

4 Global Anxiety: SCL-10, Mini Social Phobia Inventory (Mini-SPIN), and the Screen for Child Anxiety Related Disorders (SCARED).

5 *p* value (two-sided) from independent sample *t*-test: ^***^p < 0.001.

The findings indicated that the non-help-seeking at-risk adolescents exhibited significantly higher depression and anxiety scores compared to controls. Furthermore, they experienced significantly lower self-esteem and social function than controls.

When analyzing the difference between groups in areas of social function (leisure time, social competence, quality of parental relationship, school belonging), the independent samples *t*-test indicated significantly lower experienced function (*p* < 0.01) in all four domains (Table 3).

**Table 3:** Comparison of different areas of social function between adolescents at-risk and controls.

	At-risk *N* = 58	Controls *N* = 69				
Social Function^1^	*M* (SD)	*M* (SD)	*t*	*p* ^2^	Cohen’s *d*	CI
Leisure time	3.2 (0.6)	3.5 (0.4)	2.79	0.006**	*d* = 0.51	[95% CI: 0.07, 0.44]
Social competence	2.6 (0.1)	3.1 (0.7)	4.43	<0.001***	*d* = 0.80	[95% CI: 0.30, 0.78]
Parental relationship quality	3.0 (0.7)	3.5 (0.5)	4.92	<0.001***	*d* = 0.89	[95% CI: 0.31, 0.72]
School belonging	2.3 (0.8)	3.2 (0.7)	5.48	<0.001***	*d* = 1.05	[95% CI: 0.52, 1.12]

1 Social Function measures in the 14-year survey included selected items about leisure activities, social competence (Self-Perceptions Profile for adolescents), parental relationship quality (Parental Relations Self-concept Scale), and school belonging (Norwegian PIRLS 2016).

2 *p* value (two-sided) from independent sample *t*-test: **p < 0.01, ***p < 0.001.

The at-risk group reported significantly fewer leisure activities, lower social competence, poorer quality of relationship with parents, and less school belonging compared to controls. The leisure time measure included, e.g., hours spent on friends, exercise, activities, gaming, and PC use. Leisure time was the only social function measure that resulted in moderate effect according to Cohen’s conventions (*d* = 0.51, *p* = 0.006). The difference between groups in social competence, parental relationship quality and school belonging had large effect sizes (*d* = 0.80, 1.05, *p* < 0.001). Overall, these findings suggest that the non-help-seeking group of adolescents at risk for psychosis, struggle significantly more within multiples areas of social functioning, than the adolescents without psychotic distress.

## Discussion

The aim of this study was to explore psychological and social functioning in a non-help-seeking group of 14-year-olds, and to compare adolescents who report psychotic symptoms with those who do not describe such challenges. Self-reports of depression, anxiety, self-esteem, and social functioning were utilized to measure concurrent psychological and social difficulties. To the best of our knowledge, this is the first non-help-seeking cohort study using self-reports to investigate the differences between 14-year-old adolescents at risk for psychosis and matched controls across a wide spectrum of psychological and social variables.

Align with previous CHR research of adolescents and young adults (Addington et al., 2017; Fusar-Poli et al., 2014; McAusland et al., 2017) and children (Lo Buglio et al., 2022), our research identified significant disparities in self-reported depression and anxiety between 14-year-olds at risk and their peers without psychotic distress, indicating that adolescents at risk, outside clinical settings, have similar psychological difficulties as youths who present for professional help. CHR research indicates that depression is the most common comorbid diagnosis among at-risk individuals (Addington et al., 2021), but high levels of general anxiety disorders (McAusland et al., 2017) and specifically social anxiety (Deng et al., 2023) are also reported, and studies show that anxiety frequently accompanies depression (McAusland et al., 2017). In general, both conditions commonly overlap, with 45.7% of individuals with major depression also experiencing one or more lifetime anxiety disorders (Kessler et al., 2015). Previous findings indicate that increased levels of depression and anxiety exacerbate the severity of psychotic symptoms (Shi et al., 2017). Also, the majority of at-risk individuals who do not progress to psychosis are likely to continue experiencing coexisting mental health conditions (Rutigliano et al., 2016). This underlines the importance of early detection and intervention, and insights from our study indicate that also non-help-seeking adolescents at risk should be incorporated in such interventions.

In general, low self-esteem is associated with various psychological symptoms and is frequently found to predict depression and anxiety among adolescents (Henriksen et al., 2017; Wiechert et al., 2023; Orth and Robins, 2013; Gu et al., 2024). However, the nature of the relation is debated (Orth and Robins, 2013; Sowislo and Orth, 2013). A meta-analysis indicated that the effect of low self-esteem on depression is significantly stronger than vice versa (Sowislo and Orth, 2013). In contrast, the relationship between anxiety and self-esteem seems more balanced, with each predicting the other (Sowislo and Orth, 2013). Our study observed significantly lower self-esteem among at-risk adolescents compared to controls. The difference may be linked to the presence of psychotic symptoms or other psychological difficulties, such as depression. Due to the small sample size, analyzing the precise nature of the relationship between self-esteem and other symptoms was statistically challenging. However, understanding how these symptoms interact is important for tailoring interventions and treatments, and necessitates further investigation in studies with larger sample sizes.

Our findings of low self-esteem in at-risk adolescents, align with existing research documenting diminished quality of life, self-esteem, and self-efficacy among children, adolescents, and young CHR adults (Lo Buglio et al., 2022; Heinze et al., 2018; Asher et al., 2013; Rusch et al., 2014). Psychosis is frequently related to personal stigma (Gerlinger et al., 2013; Lien et al., 2018; Fond et al., 2023) and reduced self-esteem (Hofer et al., 2023; Ciufolini et al., 2015), a phenomenon observed not only in established psychosis but also among adults identified at ultra-high risk for psychosis (Bemrose et al., 2021). Notably, low self-esteem emerges as a critical impediment to recovery, demonstrating a correlation with the severity of symptoms (Hofer et al., 2023). Moreover, lower self-esteem seems largely connected to negative views on social functioning and social skills (Benavides et al., 2018).

When examining social functioning, we found large impairments among adolescents at risk compared to their non-psychotic peers. This suggests that this vulnerable group faces social challenges similar to those at CHR (Lo Buglio et al., 2022; Addington et al., 2021; Ballon et al., 2007; Shim et al., 2008; Braun et al., 2022; Devoe et al., 2019). In general, research employs a diverse range of indicators to assess the dimension of social functioning, with a predominant focus on leisure activities and peer relationships. In our study, we evaluated four domains: leisure time, social competence, quality of parental relationships, and school belonging. We found that adolescents at risk displayed significantly more difficulties than controls in all assessed domains. Concerning leisure activities, our results revealed that at-risk adolescents spend less time with friends, engage in fewer physical activities, have fewer hobbies, participate in organized activities less frequently, and allocate significantly more time to gaming and PC use. Furthermore, deficits in social competence suggest that they face greater challenges in forming friendships and maintaining relationships compared to their peers without psychotic symptoms. At home, our findings indicated diminished quality of parental relationships. This result is congruent with previous research among individuals at CHR (Thompson et al., 2015). The inclusion of school belonging in at-risk research is infrequent. Nonetheless, school belonging plays a critical role in mitigating loneliness, and promoting social inclusion, prosocial behavior and psychosocial well-being (Allen et al., 2021). Facilitating academic support via social networks and enhancing the sense of school belonging are important strategies to strengthen hope and optimism among youth facing psychological or social adversities (Sulimani-Aidan and Melkman, 2022).

Our research expands upon insights from previous studies to non-help-seeking populations of at-risk adolescents, highlighting that the vulnerability to psychological and social challenges is not confined to clinical populations. The consistency of these challenges across diverse populations of adolescents and young adults indicates a more expansive phenotype of psychosis risk, encompassing both social and emotional dimensions. Moreover, self-report screening instruments offer promising possibilities for early identification of psychosis risk. Evidence suggests that both adolescent and parental reports are predictive of clinical assessments, with an amplified predictive power when these reports are combined (Thompson et al., 2014). This highlights the value of integrating both self- and parent-reported identification of youths at risk for psychosis, as well as including school and community surveys to reach adolescents who are outside the healthcare system. Underscoring the necessity of including adolescents self-reports is that parental reports are found less effective than self-reports in identifying concurrent social and behavioral difficulties, particularly in the context of predicting psychosis (Simeonova et al., 2011). Early identification and intervention are critical, as evidenced by research indicating that timely interventions are both effective and cost-efficient for individuals exhibiting psychotic symptoms, even among those who do not progress to psychosis (Allan et al., 2021). Such interventions can reduce the duration of untreated psychosis, enhance treatment adherence, mitigate social decline, lower the probability of transitioning to psychosis, reduce symptom severity and distress, and improve overall quality of life (Allan et al., 2021).

Despite the clear benefits of early intervention, a significant gap remains in the early detection of individuals at risk, with very few being identified prior to the onset of psychosis (McGorry and Mei, 2018). Among adolescents, findings support that many avoid seeking professional help (Nordgaard et al., 2021; Allan et al., 2021). Van Os and Guloksuz (2017) suggest a shift in focus toward broader symptom assessments, rather than an exclusive emphasis on psychotic symptoms. A more practical and efficient strategy may be to adopt a public health approach that prioritizes accessible, low-stigma, optimistic, and youth-aligned interventions (McGorry and Mei, 2018). Our study demonstrates the possibility of reaching a large number of at-risk adolescents through self-report instruments. Enhanced early detection efforts can facilitate appropriate interventions, thereby improving outcomes for at-risk individuals, in line with suggestions from Fusar-Poli et al. (2020).

### Clinical and social implications

The identification of profound psychological and social difficulties in non-help-seeking at-risk adolescents underscores the need for broader screening and intervention strategies beyond traditional clinical settings. Schools and community settings may serve as effective platforms for early identification, leveraging self-report measures similar to those used in our study. By intervening early, we can potentially mitigate the progression of symptoms and improve long-term outcomes for young people at risk for psychosis.

### Limitations and future directions

Our study’s reliance solely on self-reported measures introduces the potential for bias in our material. However, as noted earlier, evidence indicates that adolescents self-reports of symptoms are predictive of symptoms assessed by clinicians, and even more so if including parents-reports (Thompson et al., 2014). Hence, the inclusion of parents-reports and/or other multimodal assessment strategies could have reduced the possibility for bias and is recommended in future research. Importantly, we wanted to reach a wide group of 14-year-olds, and the use of data from a large cohort study such as MoBa, gave us a unique opportunity to explore individuals often difficult to reach.

Additionally, most study participants were girls, comprising over 86% in both at-risk and control groups. Since groups were sex-matched, this should not have influenced the results. In general, females are more likely to participate in online surveys (Keusch, 2015; Woodall et al., 2010). Moreover, adolescent girls in PLEs studies report more psychotic-like symptoms than boys, but importantly, the effects on depression and anxiety seem to be similar (Stainton et al., 2021).

The specific cut-off used to define our at-risk group, while methodologically sound, may not capture the full spectrum of psychosis risk. The strict cut-off capturing only the top 0.4% scorers on psychotic symptoms was chosen to avoid the risk of including false positives. Thus, it was imperative to include only the adolescents with high levels of psychotic symptoms. Moreover, the carefully chosen items from both the CAPE and the EASE are found to capture psychosis risk by predicting conversion to psychosis (Bukenaite et al., 2017; Nelson et al., 2012; Jaya et al., 2021; Nunez et al., 2021; Sengutta et al., 2021; Vaernes et al., 2019; Svendsen et al., 2018).

Finally, our small sample size made it difficult to accurately analyze the relationship between self-esteem and other symptoms. Nonetheless, understanding the interactions between these symptoms is crucial for adapting interventions and treatments, highlighting the need for further research with larger sample sizes.

## Conclusion

This study represents an exploration into the psychological and social challenges faced by non-help-seeking 14-year-olds at risk for psychosis. By leveraging self-reported data from the Norwegian Mother, Father, and Child Cohort Study (MoBa), we detected significant psychological distress and functional impairments experienced by this group. Our findings reveal similarities with research from CHR populations, particularly of elevated rates of depression and anxiety, as well as reduced self-esteem and social function. These findings not only validate the broader spectrum of psychosis risk but also highlight the need for inclusive screening and intervention strategies that extend beyond traditional clinical settings.

Our study supports a broader perspective on psychosis risk assessment and intervention, toward public health strategies. Self-report instruments addressing a wide spectrum of symptoms and challenges may be distributed through schools or primary healthcare settings. This can improve early detection of at-risk adolescents, but may also offer timely, effective support and interventions, aimed at improving psychosocial well-being and resilience among these vulnerable youths.

## Data Availability

Data from the Norwegian Mother, Father and Child Cohort Study and the Medical Birth Registry of Norway used in this study are managed by the national health register holders in Norway (Norwegian Institute of Public Health) and can be made available to researchers, provided approval from the Regional Committees for Medical and Health Research Ethics (REC), compliance with the EU General Data Protection Regulation (GDPR) and approval from the data owners. Researchers who want access to datasets for replication should apply through helsedata.no. Access to datasets requires approval from The Regional Committee for Medical and Health Research Ethics in Norway and an agreement with MoBa. Requests to access the datasets should be directed to https://www.fhi.no/en/ch/studies/moba/for-forskere-artikler/viktige-dokumenter-for-moba-forskere/.

## References

[ref1] Addington J. Farris M. S. Liu L. Cadenhead K. S. Cannon T. D. Cornblatt B. A.. (2021). Depression: an actionable outcome for those at clinical high-risk. Schizophr. Res. 227, 38–43. doi: 10.1016/j.schres.2020.10.001, PMID: 33129638 PMC7854482

[ref2] Addington J. Piskulic D. Liu L. Lockwood J. Cadenhead K. S. Cannon T. D.. (2017). Comorbid diagnoses for youth at clinical high risk of psychosis. Schizophr. Res. 190, 90–95. doi: 10.1016/j.schres.2017.03.043 28372906 PMC5731830

[ref3] Addington J. Santesteban-Echarri O. Braun A. Lecomte T. (2021). Assessing social functioning in youth at clinical high-risk for psychosis. Schizophr. Res. 228, 188–189. 33444935 10.1016/j.schres.2020.12.017

[ref4] Allan S. M. Hodgekins J. Beazley P. Oduola S. (2021). Pathways to care in at-risk mental states: a systematic review. Early Interv. Psychiatry 15, 1092–1103. doi: 10.1111/eip.13053, PMID: 33047505

[ref5] Allen K.-A. Slaten C. D. Arslan G. Roffey S. Craig H. Vella-Brodrick D. A. (2021). “School belonging: the importance of student and teacher relationships” in The Palgrave handbook of positive education. eds. Kern M. L. Wehmeyer M. L. (Cham: Springer International Publishing), 525–550.

[ref6] Angold A. Costello E. Messer S. Pickles A. Winder F. Silver D. (1995). The development of a questionnaire for use in epidemiological studies of depression in children and adolescents. Int. J. Methods Psychiatr. Res. 5, 237–249.

[ref7] Asher L. Zammit S. Sullivan S. Dorrington S. Heron J. Lewis G. (2013). The relationship between psychotic symptoms and social functioning in a non-clinical population of 12year olds. Schizophr. Res. 150, 404–409. doi: 10.1016/j.schres.2013.08.031 24021878

[ref8] Ballon J. S. Kaur T. Marks I. I. Cadenhead K. S. (2007). Social functioning in young people at risk for schizophrenia. Psychiatry Res. 151, 29–35. doi: 10.1016/j.psychres.2006.10.012 17383739 PMC3065359

[ref9] Bemrose H. V. Akande I. O. Cullen A. E. (2021). Self-esteem in individuals at ultra-high risk for psychosis: a systematic review and meta-analysis. Early Interv. Psychiatry 15, 775–786. doi: 10.1111/eip.13034 32860493

[ref10] Benavides C. Brucato G. Kimhy D. (2018). Self-esteem and symptoms in individuals at clinical high risk for psychosis. J. Nerv. Ment. Dis. 206, 433–438. doi: 10.1097/nmd.0000000000000824 29781891 PMC5980752

[ref11] Birmaher B. Brent D. A. Chiappetta L. Bridge J. Monga S. Baugher M. (1999). Psychometric properties of the screen for child anxiety related emotional disorders (SCARED): a replication study. J. Am. Acad. Child Adolesc. Psychiatry 38, 1230–1236. doi: 10.1097/00004583-199910000-00011, PMID: 10517055

[ref12] Birmaher B. Khetarpal S. Brent D. Cully M. Balach L. Kaufman J.. (1997). The screen for child anxiety related emotional disorders (SCARED): scale construction and psychometric characteristics. J. Am. Acad. Child Adolesc. Psychiatry 36, 545–553. doi: 10.1097/00004583-199704000-00018, PMID: 9100430

[ref13] Blakemore S. J. (2019). Adolescence and mental health. Lancet 393, 2030–2031. doi: 10.1016/S0140-6736(19)31013-X 31106741

[ref14] Braun A. Santesteban-Echarri O. Cadenhead K. S. Cornblatt B. A. Granholm E. Addington J. (2022). Bullying and social functioning, schemas, and beliefs among youth at clinical high risk for psychosis. Early Interv. Psychiatry 16, 281–288. doi: 10.1111/eip.13157 33938145

[ref15] Bukenaite A. Stochl J. Mossaheb N. Schafer M. R. Klier C. M. Becker J.. (2017). Usefulness of the CAPE-P15 for detecting people at ultra-high risk for psychosis: psychometric properties and cut-off values. Schizophr. Res. 189, 69–74. doi: 10.1016/j.schres.2017.02.017, PMID: 28254243

[ref16] Ciufolini S. Morgan C. Morgan K. Fearon P. Boydell J. Hutchinson G.. (2015). Self esteem and self agency in first episode psychosis: ethnic variation and relationship with clinical presentation. Psychiatry Res. 227, 213–218. doi: 10.1016/j.psychres.2015.03.030, PMID: 25868868

[ref17] Cleverley K. Foussias G. Ameis S. H. Courtney D. B. Goldstein B. I. Hawke L. D.. (2024). The Toronto adolescent and youth cohort study: study design and early data related to psychosis spectrum symptoms, functioning, and suicidality. Biol. Psychiatry Cogn. Neurosci. Neuroimag. 9, 253–264. doi: 10.1016/j.bpsc.2023.10.011, PMID: 37979943

[ref18] Colizzi M. Lasalvia A. Ruggeri M. (2020). Prevention and early intervention in youth mental health: is it time for a multidisciplinary and trans-diagnostic model for care? Int. J. Ment. Heal. Syst. 14:23. doi: 10.1186/s13033-020-00356-9 PMC7092613 32226481

[ref19] Connor K. M. Kobak K. A. Churchill L. E. Katzelnick D. Davidson J. R. (2001). Mini-SPIN: a brief screening assessment for generalized social anxiety disorder. Depress. Anxiety 14, 137–140. doi: 10.1002/da.1055 11668666

[ref20] Deng W. Addington J. Bearden C. E. Cadenhead K. S. Cornblatt B. A. Mathalon D. H.. (2023). Characterizing sustained social anxiety in individuals at clinical high risk for psychosis: trajectory, risk factors, and functional outcomes. Psychol. Med. 53, 3644–3651. doi: 10.1017/S0033291722000277, PMID: 35144716 PMC10277760

[ref21] Devoe D. J. Farris M. S. Townes P. Addington J. (2019). Interventions and social functioning in youth at risk of psychosis: a systematic review and meta-analysis. Early Interv. Psychiatry 13, 169–180. doi: 10.1111/eip.12689 29938910

[ref23] Fond G. Vidal M. Joseph M. Etchecopar-Etchart D. Solmi M. Yon D. K.. (2023). Self-stigma in schizophrenia: a systematic review and meta-analysis of 37 studies from 25 high- and low-to-middle income countries. Mol. Psychiatry 28, 1920–1931. doi: 10.1038/s41380-023-02003-4 36890299

[ref24] Fonseca-Pedrero E. Ortuño-Sierra J. Chocarro E. Inchausti F. Debbané M. Bobes J. (2017). Psychosis risk screening: validation of the youth psychosis at-risk questionnaire – brief in a community-derived sample of adolescents. Int. J. Methods Psychiatr. Res. 26:e1543. doi: 10.1002/mpr.1543, PMID: 27790784 PMC6877222

[ref25] Fusar-Poli P. Borgwardt S. Bechdolf A. Addington J. Riecher-Rossler A. Schultze-Lutter F.. (2013). The psychosis high-risk state: a comprehensive state-of-the-art review. JAMA Psychiatry 70, 107–120. doi: 10.1001/jamapsychiatry.2013.269 23165428 PMC4356506

[ref26] Fusar-Poli P. Nelson B. Valmaggia L. Yung A. R. McGuire P. K. (2014). Comorbid depressive and anxiety disorders in 509 individuals with an at-risk mental state: impact on psychopathology and transition to psychosis. Schizophr. Bull. 40, 120–131. doi: 10.1093/schbul/sbs136 23180756 PMC3885287

[ref27] Fusar-Poli P. Salazar de Pablo G. Correll C. U. Meyer-Lindenberg A. Millan M. J. Borgwardt S.. (2020). Prevention of psychosis: advances in detection, prognosis, and intervention. JAMA Psychiatry 77, 755–765. doi: 10.1001/jamapsychiatry.2019.4779 32159746

[ref28] Gerlinger G. Hauser M. De Hert M. Lacluyse K. Wampers M. Correll C. U. (2013). Personal stigma in schizophrenia spectrum disorders: a systematic review of prevalence rates, correlates, impact and interventions. World Psychiatry 12, 155–164. doi: 10.1002/wps.20040, PMID: 23737425 PMC3683268

[ref29] Gu H. Zhang P. Li J. (2024). The effect of self-esteem on depressive symptoms among adolescents: the mediating roles of hope and anxiety. Human. Soc. Sci. Commun. 11:932. doi: 10.1057/s41599-024-03249-1

[ref30] Harter S. (1988). Manual for the self-perception profile for adolescents. Denver, CO: University of Denver.

[ref31] Harter S. (2012). Self-perception profile for adolescents: Manual and questionnaires. Denver: University of Denver, Department of Psychology.

[ref32] Heinze K. Lin A. Nelson B. Reniers R. Upthegrove R. Clarke L.. (2018). The impact of psychotic experiences in the early stages of mental health problems in young people. BMC Psychiatry 18:214. doi: 10.1186/s12888-018-1767-y, PMID: 29954377 PMC6025824

[ref33] Henriksen I. O. Ranøyen I. Indredavik M. S. Stenseng F. (2017). The role of self-esteem in the development of psychiatric problems: a three-year prospective study in a clinical sample of adolescents. Child Adolesc. Psychiatry Ment. Health 11:68. doi: 10.1186/s13034-017-0207-y 29299058 PMC5747942

[ref34] Hofer A. Biedermann F. Kaufmann A. Kemmler G. Pfaffenberger N. M. Yalcin-Siedentopf N. (2023). Self-esteem in stabilized individuals with chronic schizophrenia: association with residual symptoms and cognitive functioning. Eur. Arch. Psychiatry Clin. Neurosci. 273, 1737–1746. doi: 10.1007/s00406-022-01538-x 36602648 PMC10713693

[ref35] Howie C. Potter C. Shannon C. Davidson G. Mulholland C. (2020). Screening for the at-risk mental state in educational settings: a systematic review. Early Interv. Psychiatry 14, 643–654. doi: 10.1111/eip.12926 31883215

[ref36] Jaya E. S. van Amelsvoort T. Bartels-Velthuis A. A. Bruggeman R. Cahn W. de Haan L.. (2021). The community assessment of psychic experiences: optimal cut-off scores for detecting individuals with a psychotic disorder. Int. J. Methods Psychiatr. Res. 30:e1893. doi: 10.1002/mpr.1893 34464487 PMC8633944

[ref37] Jongsma H. E. Turner C. Kirkbride J. B. Jones P. B. (2019). International incidence of psychotic disorders, 2002-17: a systematic review and meta-analysis. Lancet Public Health 4, e229–e244. doi: 10.1016/S2468-2667(19)30056-8 31054641 PMC6693560

[ref38] Kessler R. C. Sampson N. A. Berglund P. Gruber M. J. Al-Hamzawi A. Andrade L.. (2015). Anxious and non-anxious major depressive disorder in the World Health Organization world mental health surveys. Epidemiol. Psychiatr. Sci. 24, 210–226. doi: 10.1017/S2045796015000189, PMID: 25720357 PMC5129607

[ref39] Keusch F. (2015). Why do people participate in web surveys? Applying survey participation theory to internet survey data collection. Manag. Rev. Quart. 65, 183–216. doi: 10.1007/s11301-014-0111-y

[ref40] Lien Y. J. Chang H. A. Kao Y. C. Tzeng N. S. Lu C. W. Loh C. H. (2018). Insight, self-stigma and psychosocial outcomes in schizophrenia: a structural equation modelling approach. Epidemiol. Psychiatr. Sci. 27, 176–185. doi: 10.1017/S2045796016000950, PMID: 27974084 PMC6998951

[ref41] Lo Buglio G. Pontillo M. Cerasti E. Polari A. Schiano Lomoriello A. Vicari S.. (2022). A network analysis of anxiety, depressive, and psychotic symptoms and functioning in children and adolescents at clinical high risk for psychosis. Front. Psych. 13:1016154. doi: 10.3389/fpsyt.2022.1016154, PMID: 36386985 PMC9650363

[ref42] Magnus P. Birke C. Vejrup K. Haugan A. Alsaker E. Daltveit A. K.. (2016). Cohort profile update: the Norwegian mother and child cohort study (MoBa). Int. J. Epidemiol. 45, 382–388. doi: 10.1093/ije/dyw029, PMID: 27063603

[ref43] Marsh H. W. Ellis L. A. Parada R. H. Richards G. Heubeck B. G. (2005). A short version of the self description questionnaire II: operationalizing criteria for short-form evaluation with new applications of confirmatory factor analyses. Psychol. Assess. 17, 81–102. doi: 10.1111/j.1467-8624.2005.00853.x 15769230

[ref44] McAusland L. Buchy L. Cadenhead K. S. Cannon T. D. Cornblatt B. A. Heinssen R.. (2017). Anxiety in youth at clinical high risk for psychosis. Early Interv. Psychiatry 11, 480–487. doi: 10.1111/eip.12274 26456932 PMC4912451

[ref45] McGlashan T. Walsh B. Woods S. (2010). The psychosis-risk syndrome: handbook for diagnosis and follow-up: Oxford University Press.

[ref46] McGorry P. D. Mei C. (2018). Early intervention in youth mental health: progress and future directions. Evid. Based Ment. Health 21, 182–184. doi: 10.1136/ebmental-2018-300060, PMID: 30352884 PMC10270418

[ref47] MoBa. (2022). Questions documentation 14-year questionnaire (Q-14aar) when the child is 14 years old. FHI The Norwegian Mother, Father, and Child Cohort Study. Available at: https://www.fhi.no/contentassets/1016188d845f4c5f8fa57266c454ad8c/instrument-documentation-q14_barn.pdf (Accessed October 31, 2022).

[ref22] MoBa. (2024). FHI, The Norwegian Mother, Father, and Child Cohort Study (MOBA). Publication_guidelines_moba [Internet]. Available at: https://www.fhi.no/globalassets/dokumenterfiler/studier/den-norske-mor-far-og-barn--undersokelsenmoba/info-til-forskere/publication_guidelines_moba.pdf

[ref48] Mohn-Haugen C. R. Mohn C. Laroi F. Teigset C. M. Oie M. G. Rund B. R. (2022). Cognitive functioning in a group of adolescents at risk for psychosis. Front. Psych. 13:1075222. doi: 10.3389/fpsyt.2022.1075222 PMC9753978 36532169

[ref49] Mohn-Haugen C. R. Møller P. Mohn C. Larøi F. Teigset C. M. Øie M. G.. (2023). Anomalous self-experiences and neurocognitive functioning in adolescents at risk for psychosis: still no significant associations found between these two vulnerability markers. Compr. Psychiatry 125:152400. doi: 10.1016/j.comppsych.2023.152400 37451231

[ref50] Nelson B. Thompson A. Yung A. R. (2012). Basic self-disturbance predicts psychosis onset in the ultra high risk for psychosis “prodromal” population. Schizophr. Bull. 38, 1277–1287. doi: 10.1093/schbul/sbs007 22349924 PMC3494062

[ref51] Nordgaard J. Nilsson L. S. Gulstad K. Buch-Pedersen M. (2021). The paradox of help-seeking behaviour in psychosis. Psychiatry Q. 92, 549–559. doi: 10.1007/s11126-020-09833-3 32821995

[ref52] Nunez D. Godoy M. I. Gaete J. Faundez M. J. Campos S. Fresno A.. (2021). The community assessment of psychic experiences-positive scale (CAPE-P15) accurately classifies and differentiates psychotic experience levels in adolescents from the general population. PLoS One 16:e0256686. doi: 10.1371/journal.pone.0256686, PMID: 34437593 PMC8389461

[ref53] Orth U. Robins R. W. (2013). Understanding the link between low self-esteem and depression. Curr. Dir. Psychol. Sci. 22, 455–460. doi: 10.1177/0963721413492763

[ref54] Parnas J. Moller P. Kircher T. Thalbitzer J. Jansson L. Handest P.. (2005). EASE: examination of anomalous self-experience. Psychopathology 38, 236–258. doi: 10.1159/000088441 16179811

[ref55] Radez J. Waite F. Izon E. Johns L. (2023). Identifying individuals at risk of developing psychosis: a systematic review of the literature in primary care services. Early Interv. Psychiatry 17, 429–446. doi: 10.1111/eip.13365 36632681 PMC10946574

[ref56] Rosenberg M. (1965). Society and the adolescent self-image. New Jersey: Princeton University Press.

[ref57] Rosenberg M. (1986). Conceiving the self. Malabar, FL: Krieger.

[ref58] Rusch N. Corrigan P. W. Heekeren K. Theodoridou A. Dvorsky D. Metzler S.. (2014). Well-being among persons at risk of psychosis: the role of self-labeling, shame, and stigma stress. Psychiatr. Serv. 65, 483–489. doi: 10.1176/appi.ps.201300169, PMID: 24382666

[ref59] Rutigliano G. Valmaggia L. R. Landi P. Frascarelli M. Cappucciati M. Sear V.. (2016). Persistence or recurrence of non-psychotic comorbid mental disorders associated with 6-year poor functional outcomes in patients at ultra high risk for psychosis. J. Affect. Disord. 203, 101–110. doi: 10.1016/j.jad.2016.05.053 27285723

[ref60] Sagatun A. Sogaard A. J. Bjertness E. Selmer R. Heyerdahl S. (2007). The association between weekly hours of physical activity and mental health: a three-year follow-up study of 15-16-year-old students in the city of Oslo, Norway. BMC Public Health 7:155. doi: 10.1186/1471-2458-7-155 17626617 PMC1955440

[ref61] Salokangas R. K. R. Ruhrmann S. von Reventlow H. G. Heinimaa M. Svirskis T. From T.. (2012). Axis I diagnoses and transition to psychosis in clinical high-risk patients EPOS project: prospective follow-up of 245 clinical high-risk outpatients in four countries. Schizophr. Res. 138, 192–197. doi: 10.1016/j.schres.2012.03.008, PMID: 22464922

[ref62] Schmider E. Ziegler M. Danay E. Beyer L. Bühner M. (2010). Is it really robust? Reinvestigating the robustness of ANOVA against violations of the normal distribution assumption. Methodol. Eur. J. Res. Methods Behav. Soc. Sci. 6, 147–151. doi: 10.1027/1614-2241/a000016

[ref63] Schultze-Lutter F. Schimmelmann B. G. Flückiger R. Michel C. (2020). Effects of age and sex on clinical high-risk for psychosis in the community. World J. Psychiatry 10, 101–124. doi: 10.5498/wjp.v10.i5.101 32477906 PMC7243619

[ref64] Seeley-Wait E. Abbott M. J. Rapee R. M. (2009). Psychometric properties of the mini-social phobia inventory. Prim. Care Companion J. Clin. Psychiatry 11, 231–236. doi: 10.4088/PCC.07m00576 19956461 PMC2781035

[ref65] Sengutta M. Karow A. Gaweda L. (2021). Anomalous self-experiences (ASE) in relation to clinical high risk for psychosis (CHRP), childhood trauma and general psychopathology among adolescent and young adult help seekers. Schizophr. Res. 237, 182–189. doi: 10.1016/j.schres.2021.09.009 34536752

[ref66] Shi J. Wang L. Yao Y. Su N. Zhan C. Mao Z.. (2017). Comorbid mental disorders and 6-month symptomatic and functioning outcomes in Chinese university students at clinical high risk for psychosis. Front. Psych. 8:8. doi: 10.3389/fpsyt.2017.00209 PMC5660058 29109690

[ref67] Shim G. Kang D.-H. Sun Chung Y. Young Yoo S. Young Shin N. Soo K. J. (2008). Social functioning deficits in Young people at risk for schizophrenia. Austral. N. Z. J. Psychiatry 42, 678–685. doi: 10.1080/00048670802203459 18622775

[ref68] Simeonova D. I. Attalla A. Trotman H. Esterberg M. Walker E. F. (2011). Does a parent-report measure of behavioral problems enhance prediction of conversion to psychosis in clinical high-risk adolescents? Schizophr. Res. 130, 157–163. doi: 10.1016/j.schres.2011.03.034 21521630 PMC3139757

[ref69] Solmi M. Soardo L. Kaur S. Azis M. Cabras A. Censori M.. (2023). Meta-analytic prevalence of comorbid mental disorders in individuals at clinical high risk of psychosis: the case for transdiagnostic assessment. Mol. Psychiatry 28, 2291–2300. doi: 10.1038/s41380-023-02029-8, PMID: 37296309 PMC10611568

[ref70] Sowislo J. F. Orth U. (2013). Does low self-esteem predict depression and anxiety? A meta-analysis of longitudinal studies. Psychol. Bull. 139, 213–240. doi: 10.1037/a0028931 22730921

[ref71] Stainton A. Chisholm K. Woodall T. Hallett D. Reniers R. Lin A.. (2021). Gender differences in the experience of psychotic-like experiences and their associated factors: a study of adolescents from the general population. Schizophr. Res. 228, 410–416. doi: 10.1016/j.schres.2021.01.008, PMID: 33556674

[ref72] Stefanis N. C. Hanssen M. Smirnis N. K. Avramopoulos D. A. Evdokimidis I. K. Stefanis C. N.. (2002). Evidence that three dimensions of psychosis have a distribution in the general population. Psychol. Med. 32, 347–358. doi: 10.1017/S0033291701005141, PMID: 11866327

[ref73] Strand N. P. von Soest T. (2008). Young in Norway–longitudinal. Documentation of design, variables, and scales. Norway: NOVA/NTNU.

[ref74] Sulimani-Aidan Y. Melkman E. (2022). School belonging and hope among at-risk youth: the contribution of academic support provided by youths' social support networks. Child Fam. Soc. Work 27, 700–710. doi: 10.1111/cfs.12918

[ref75] Svendsen I. H. Oie M. G. Moller P. Nelson B. Melle I. Haug E. (2018). Stability in basic self-disturbances and diagnosis in a first treated psychosis: a seven year follow-up study. Schizophr. Res. 202, 274–280. doi: 10.1016/j.schres.2018.07.011, PMID: 30007869

[ref76] Tambs K. (2004). “Valg av spørsmål til kortversjoner av etablerte psykometriske instrumenter” in Ubevisst sjeleliv og bevisst samfunnsliv. Psykisk helse i en sammenheng. (Unconcious life of the soul and concious social life. Mental health in a relation. Festschrift for tom Sørensen at his 60 years anniversary), editor. Festskrift til tom Sørensen på hans 60-års dag. eds. Sandanger I. Ingebrigtsen G. Nygård J. F. Sørgaard K. W. (Nittedal: Nordkyst Psykiatrisk AS), 217–229.

[ref77] Tambs K. Røysamb E. (2014). Selection of questions to short-form versions of original psychometric instruments in MoBa. Norsk Epidemiol. 24, 195–203. doi: 10.5324/nje.v24i1-2.1822

[ref78] Thapa A. Cohen J. Guffey S. Higgins-D’Alessandro A. (2013). A review of school climate research. Rev. Educ. Res. 83, 357–385. doi: 10.3102/0034654313483907

[ref79] Thompson E. Kline E. Ellman L. M. Mittal V. Reeves G. M. Schiffman J. (2015). Emotional and behavioral symptomatology reported by help-seeking youth at clinical high-risk for psychosis. Schizophr. Res. 162, 79–85. doi: 10.1016/j.schres.2015.01.023 25638728

[ref80] Thompson E. Kline E. Reeves G. Pitts S. C. Bussell K. Schiffman J. (2014). Using parent and youth reports from the behavior assessment system for children, second edition to identify individuals at clinical high-risk for psychosis. Schizophr. Res. 154, 107–112. doi: 10.1016/j.schres.2014.02.009 24630261

[ref81] Tikkanen V. Siira V. Wahlberg K.-E. Hakko H. H. Läksy K. Roisko R.. (2019). Adolescent social functioning in offspring at high risk for schizophrenia spectrum disorders in the Finnish adoptive family study of schizophrenia. Schizophr. Res. 215, 293–299. doi: 10.1016/j.schres.2019.10.013 31699628

[ref82] Tikkanen V. Siira V. Wahlberg K.-E. Hakko H. Myllyaho T. Läksy K.. (2022). Deficits in adolescent social functioning, dysfunctional family processes and genetic risk for schizophrenia spectrum disorders as risk factors for later psychiatric morbidity of adoptees. Psychiatry Res. 316:114793. doi: 10.1016/j.psychres.2022.114793, PMID: 35987066

[ref83] Vaernes T. G. Rossberg J. I. Moller P. (2019). Anomalous self-experiences are strongly associated with negative symptoms in a clinical high-risk for psychosis sample. Compr. Psychiatry 93, 65–72. doi: 10.1016/j.comppsych.2019.07.003 31351243

[ref84] Van Os J. Guloksuz S. (2017). A critique of the “ultra-high risk” and “transition” paradigm. World Psychiatry 16, 200–206. doi: 10.1002/wps.20423 28498576 PMC5428198

[ref85] Wang M.-T. Degol J. L. (2016). School climate: a review of the construct, measurement, and impact on student outcomes. Educ. Psychol. Rev. 28, 315–352. doi: 10.1007/s10648-015-9319-1

[ref86] WHO. Mental health of adolescents. Available at: https://www.who.int/news-room/fact-sheets/detail/adolescent-mental-health#:~:text=It%20is%20estimated%20that%203.6%25%20of%2010%E2%80%9314-year-olds%20and,adolescents%20aged%2010%E280931420years%2C%20and%202.825%20of%2015%E2809319-year-olds (Accessed November 21, 2021).

[ref87] Wichstraum L. (1995). Harter's self-perception profile for adolescents: reliability, validity, and evaluation of the question format. J. Pers. Assess. 65, 100–116. 7643294 10.1207/s15327752jpa6501_8

[ref88] Wiechert S. van Bockstaele B. Vertregt M. van Marwijk L. Maric M. (2023). Explicit and implicit self-esteem and their associations with symptoms of anxiety and depression in children and adolescents. Eur. J. Dev. Psychol. 20, 823–838. doi: 10.1080/17405629.2023.2216447

[ref89] Wigman J. T. van Nierop M. Vollebergh W. A. Lieb R. Beesdo-Baum K. Wittchen H.-U.. (2012). Evidence that psychotic symptoms are prevalent in disorders of anxiety and depression, impacting on illness onset, risk, and severity—implications for diagnosis and ultra–high risk research. Schizophr. Bull. 38, 247–257. doi: 10.1093/schbul/sbr196, PMID: 22258882 PMC3283146

[ref90] Woodall A. Morgan C. Sloan C. Howard L. (2010). Barriers to participation in mental health research: are there specific gender, ethnicity and age related barriers? BMC Psychiatry 10:103. doi: 10.1186/1471-244X-10-103 21126334 PMC3016310

[ref91] Woods S. W. Addington J. Cadenhead K. S. Cannon T. D. Cornblatt B. A. Heinssen R.. (2009). Validity of the prodromal risk syndrome for first psychosis: findings from the north American Prodrome longitudinal study. Schizophr. Bull. 35, 894–908. doi: 10.1093/schbul/sbp027, PMID: 19386578 PMC2728816

[ref92] Wu Z. Liu Z. Zou Z. Wang F. Zhu M. Zhang W.. (2021). Changes of psychotic-like experiences and their association with anxiety/depression among young adolescents before COVID-19 and after the lockdown in China. Schizophr. Res. 237, 40–46. doi: 10.1016/j.schres.2021.08.020, PMID: 34481204 PMC8437585

[ref93] Yoon Y. Eisenstadt M. Lereya S. T. Deighton J. (2023). Gender difference in the change of adolescents' mental health and subjective wellbeing trajectories. Eur. Child Adolesc. Psychiatry 32, 1569–1578. doi: 10.1007/s00787-022-01961-4 35246720 PMC8896070

